# Serum insulin-like growth factor 1 (IGF-1) in multiple sclerosis: relation to cognitive impairment and fatigue

**DOI:** 10.1186/s41983-018-0026-y

**Published:** 2018-09-15

**Authors:** Rania S. Nageeb, Noha A. Hashim, Amal Fawzy

**Affiliations:** 1 Department of Neurology, Faculty of Medicine, Zagazig University, Sharkia, Egypt; 2 Department of Biochemistry, Faculty of Medicine, Zagazig University, Sharkia, Egypt

**Keywords:** Insulin-like growth factor-1, Cognitive impairment, Multiple sclerosis, Fatigue

## Abstract

**Background:**

Multiple sclerosis (MS) is a chronic demyelinating central nervous system (CNS) disease. Changes in insulin growth factor 1 (IGF-1) input to the brain can affect survival of myelin and CNS cells. The study aims to investigate the relation of serum IGF-1 levels with cognitive impairment and fatigue in MS patients.

**Methods:**

This study was conducted on 46 MS patients and 46 healthy controls. All participants were subjected to clinical assessment, serum IGF-1 levels, expanded disability status scale (EDSS), modified fatigue impact scale (MFIS), and Montreal cognitive assessment (MoCA) scale.

**Results:**

There was no significant difference between patients and controls regarding serum IGF-1 levels (*P* = 0.19). However, low serum levels of IGF-1 have significantly greater odds for fatigue (*P* = 0.002) and cognitive impairment (*P* < 0.001). Also, serum IGF-1 levels have a significant negative correlation with MFIS (*r* = − 0.701 and *P* < 0.001) and a significant positive correlation with MoCA scale (*r* = + 0.84 and *P* < 0.001).

**Conclusions:**

The results, specifically that low levels of serum IGF-1 was associated with cognitive impairment and fatigue in MS, suggest that IGF-I may be involved in the pathogenesis of cognitive deficits and fatigue in MS disease.

## Background

Multiple sclerosis (MS) is a chronic immune-mediated disease with neuronal demyelination and axonal injury (Patejdl et al. 2016). It is associated with disability and lower quality of life. The most apparent source of disability in MS patients is the physical and mental impacts of the disease which have frequently been neglected (Moore et al. 2012).

Cognitive impairment affects up to 43–70% of MS patients. It has a complicated neuroanatomic and pathophysiologic background (Grzegorski and Losy 2017). Vital cognitive domains which are mainly affected are the speed of information processing, memory, attention, and executive functions (Tag El-din et al. 2016).

Fatigue often presents in the earliest phase of the disease and affects up to 70% of MS patients. It is described by nearly half of patients as the worst disabling symptoms. Fatigue in MS patients is multidimensional including both physical and mental aspects. It can be primary or secondary to sleep disturbances, depression, or the use of immunomodulating therapies (Catalan et al. 2011). Fatigue was negatively correlated with disease duration and with cognitive impairment (Effata et al. 2016).

The pathogenesis of primary fatigue in MS is still understood, but recent study suggested the central origin hypothesis. Diffuse cortical damage circumscribed lesions in frontal, parietotemporal lobes, basal ganglia, and thalamus as well as compensatory neuroplasticity which can be seen in the CNS of MS patients. Also, there is a growing evidence supporting the role of the active autoimmunity comes from the effect of the recent immunotherapeutics which can ameliorate and stabilize fatigue (Patejdl et al. 2016) Moreover, El-Tamawy and colleagues (2016) found that primary fatigue contributed to cognitive impairment in Egyptian MS patients.

Insulin-like growth factor 1 (IGF-1) is a complex peptide hormone produced in multiple tissue including the brain. IGF-1 is a potent survival factor for oligodendrocyte and has a potent myelinogenic capacity. Its main function is to enhance neuronal survival, inhibit apoptosis, and increase the number of lymphocytes as well as stimulate their function (Ghassan et al. 2017).

To our knowledge, no previous study has investigated the association between fatigue and cognitive function in multiple sclerosis and serum IGF-1 value. Therefore, the current case control study was designed to investigate whether in MS patients circulating serum levels of IGF-1 has been found to be related to cognitive impairment and fatigue.

## Methods

This case-control study was conducted in the Neurology Department, Zagazig University Hospitals, on 46 patients (22 male, 24 female) with clinically definite multiple sclerosis (MS). Their ages (mean ± SD)/years were 35.11 ± 4.73.

*Ethical consideration*: A written consent was taken from all of the participants or their relatives before enrollment. The study was approved by the Institutional Ethics Committee of the Faculty of Medicine, Zagazig University (ZU-IRB#4730\ 24-12-2017).

*Inclusion criteria*: Patients who were diagnosed to have definite MS and were classified to relapsing remittent (RR) or progressive course according to the McDonald criteria were included (Polman et al. 2011).

Patients with pregnancy and lactation were excluded. Other criteria for exclusion were the occurrence of relapses or using of corticosteroid agents during the study or within the past 4 weeks, patients who were diagnosed as MS for less than 2 years and those with regular intake of antidepressants. Patients with history of neurological disorder other than MS, history of head trauma, patients with drug or alcohol abuse, patients with hearing loss or vision loss, and those with concomitant medical diseases that may affect cognitive functions, e.g., hypothyroidism, hepatic failure, and renal failure are also excluded from the study.

A control group was also recruited comprising 46 apparently healthy subjects (19 males, 27 females) from relatives of patients during the same study period consecutively; they were matched for age, gender, ethnicity, and menstrual status for females. Their ages (mean ± SD)/years were 33.19 ± 5.94.

At baseline, the patients were subjected to complete history taking (with special stress on onset, course of disease, duration, number of attacks, and type of treatment); education level was measured as the total number of years of education of each patient (illiterate and education ≤ 12 years or > 12 years). Complete general and neurological examination and the clinical disability were assessed by the expanded disability status scale (EDSS) which provides a total score on a scale that ranges from 0 (normal examination) to 10 (death from MS) (Kurtzke 1983). Fatigue was evaluated according to the modified fatigue impact scale (MFIS). The MFIS contains only 21 items and offers a more multidimensional assessment: physical (MFIS physical subscale contains 9 items), cognitive (MFIS cognitive subscale contains 10 items), and psychosocial functioning (MFIS psychogenic subscale contains 2 items). The total score is the sum of the 21 items. The score of 38 or more was defined for fatigued (Pellicano et al. 2010).

Cognition was assessed using the Arabic version of the Montreal cognitive assessment (MoCA) scale (Rahman and El Gaafary 2009). The total score is 30, and cutoff scores for cognitive impairment have been defined as follows: A score of 26 and above is defined as normal and a score ≤ 26 is defined as cognitive impairment. In those with an educational level lower than 12 years, one unit will be added to their score. The Hamilton depression scale (Hamilton 1980) is used to exclude patients with depression.

All cases underwent brain MRI after triple-dose gadolinium diethylene-triaminepenta-acetic acid.

*Laboratory investigations*: Serum samples from patients and controls were collected after overnight fasting and then centrifuged and stored at − 80 °C. Serum insulin-like growth factor 1 (IGF-1) levels were quantified in an ELISA technique (Cleveland, OH, USA). The quantification was performed with IMMULITE 2000 IGF-1 kits (Siemens, Los Angeles, CA, USA); this kit has a sensitivity of 20 ng/mL.

Also a series of routine laboratory investigations were done to exclude other causes of cognitive deficits due to system failure or other diseases, e.g., complete blood count, renal function tests, liver function tests, blood glucose, ESR, serum electrolytes, T3, T4, and anti-nuclear antibody (ANA) when indicated.

### Statistical analysis

All the statistical tests were done using Statistical Package for the Social Sciences (SPSS version, 22) (Levesque 2007). Qualitative data were presented as number (*N*) and percentage (%). Quantitative data were expressed as mean (M) ± standard deviation (SD). Chi-square (*χ*^2^) test was used to compare between groups. The association between a particular blood and clinical parameter with fatigue and cognitive affection was assessed by odds ratios (ORs) and their 95% confidence intervals (95% CIs). Spearman’s correlation analysis was done between selected study parameters. To examine the associations between serum insulin-like growth factor 1 (IGF-1) levels and the frequency of fatigue and cognitive affection in patients, subjects were divided according to IGF-1 quartiles (Q1–Q4): Q1 > 90 ng/mL, Q2 90–120 ng/mL, Q3 121–190 ng/mL, and Q4 > 191 ng/mL. All tests were two-sided, and *P* < 0.05 was considered statistically significant.

## Results

Forty-six (22 males and 24 females) MS patients besides the 46 (19 males and 27 females) controls were included in the present study. Their ages (mean ± SD)/years were 35.11 ± 4.73 and 33.19 ± 5.94 years respectively.

### Characteristic of the patient group

This study includes 12 (26.09%) MS patients presented with progressive course, and 34 (73.91%) patients with relapsing remittent (RR) MS with mean expanded disability status scale (EDSS) score of patients was 4.67 ± 2.58. The disease duration was ≤ 5 years in 67.4% of patients and > 5 years in 32.6% of patients. Regarding the number of attacks, 58.7% of patients had > 3 attacks and 41.3% of patients had ≤ 3 attacks. Twenty-eight patients (60.9%) were not on any immunomudulatory therapy, and 39.1% of patients were receiving specific treatment (6 patients on fingolimod and 12 patients on interferon β (INF-β)).

The patients and control groups had no statistically significant difference regarding age, gender, or years of education. Patients have a statistically significant lower MoCA score and a statistically significant higher MFIS score in comparison to the control group (*P* < 0.001). When the three components of the MFIS score (physical, cognitive, and psychosocial) were analyzed, all three were significantly higher in patients than in controls. Regarding IGF1, the mean serum level in the patient group (138.37 ± 53.65) was lower than in the control group (151.77 ± 42.92) but with no statistically significant difference (*P* = 0.19) (Table 1).

**Table 1:** Demographic data, clinical characteristics, and scales as well as serum IGF-1 levels of the studied groups

Variable	Patients (46)	Controls (46)	*P* value
Age, years	35.11 ± 4.73	33.19 ± 5.94	0.3
≤ 35	25 (54.3%)	30 (65.2%)	
> 35	21 (45.7%)	16 (34.8%)	
Gender			
Male	22 (47.8%)	19 (41.3%)	0.53
Female	24 (52.2%)	27 (58.7%)	
Education			
Illiterate	5 (10.9%)	7 (15.2%)	0.5
< 12 years	10 (21.7%)	12 (26.1%)	0.6
> 12 years	31 (67.4%)	27 (58.7%)	0.4
Disease course			
Progressive	12 (26.09%)		
RR	34 (73.91%)		
EDSS	4.67 ± 2.58		
Mild–moderate (0–5.5)	26 (56.5%)		
Severe (6–9.5)	20 (43.5%)	–	–
Disease duration			
≤ 5 years	31 (67.4%)	–	
> 5 years	15 (32.6%)		–
Number of attacks			
> 3	27 (58.7%)	–	
≤ 3	19 (41.3%)		–
Treatment			
Yes	18 (39.1%)		
No	28 (60.9%)	–	–
Montreal cognitive scale	25.72 ± 3.08	28.59 ± 0.98	< 0.001*
MoCA score > 26	22 (47.8%)	46 (100%)	
MoCA score≤ 26	24 (52.2%)	–	
MFIS	68.15 ± 10.3	45.23 ± 23.14	< 0.001*
MFIS psychogenic subscale	6.15 ± 1.29	3.81 ± 2.43	< 0.001*
MFIS physical subscale	32.11 ± 2.24	21 .54 ± 9.62	< 0.001*
MFIS cognitive subscale	35.04 ± 3.39	19.76 ± 12.75	< 0.001*
Serum IGF-1 levels (ng/mL)	138.37 ± 53.65	151.77 ± 42.92	0.19

*Significant

*EDSS* expanded disability status scale, *RR* relapsing-remitting, *MFIS* modified fatigue impact scale, *IGF-1* insulin-like growth factor 1

The frequency of fatigue (MFIS score ≥ 38) was significantly higher in MS patients with age > 35, EDSS ≥ 6, disease duration > 5 years, and number of relapse > 3. Regarding serum IGF-1 levels, the Q1 and Q2 groups have significantly greater odds for fatigue (OR 6.1, 95% CI (4.7 ± 8.42), *P* = 0.002) (OR 8.9, 95% CI (4.8 ± 16.6), *P* = 0.003) respectively while the odds for Q3 group for fatigue were lower (OR 2.3, 95% CI (0.6 ± 18.8), *P* = 0.14). Cognitive affection (MoCA score ≤ 26) was significantly more frequent in MS patients with age > 35 years, EDSS ≥ 6, disease duration > 5 years, and relapse attacks > 3 and in MS patients without specific treatment. For serum IGF-1 levels, the Q1 and Q2 groups had significantly greater odds to be with cognitive impairment (OR 8.43, 95% CI (2.4 ± 4.9), *P* < 0.001 and OR 3.01, 95% CI (0.3 ± 1.2), *P* = 0.03), while the odds for the Q3 group was OR 2.9, 95% CI (0.59 ± 1.4), *p* = 0.7 (Table 2).

**Table 2:** Risk factors of fatigue and cognitive impairment among MS patients

Variable	Fatigue		Cognitive impairment	
	(MFIS score ≥ 38)		(MOCA score ≤ 26)	
	(*N*, %) (25 patients, 54.3%)		(*N*, %) (24 patients, 52.2%)	
	OR	*P*	OR	*P*
Age, years				
> 35	12.8 (3.15 ± 52.7)	< 0.001*	16.9 (3.93 ± 73.57)	< 0.001*
≤ 35	1		1	
Gender				
Male	1	0.98	1	0.78
Female	1.02 (0.32 ± 3.24)		1.2 (0.37 ± 3.77)	
Disease course				
Progressive	1	0.76	1	0.76
RR	13.9 (0.5 ± 9.2)		25 (0.2 ± 11.2)	
EDSS				
0–5.5	1	< 0.001*	1	0.03*
6–9.5	24.43 (4.47 ± 13.53)		20.8 (2.1 ± 18.9)	
Disease duration				
≤ 5 years	1	0.003*	1	0.004*
> 5 years	25.5 (2.94 ± 220.20		11.80 (2.25 ± 62.11)	
Number of attacks				
≤ 3	1	0.002*	1	0.001*
> 3	10.66 (2.45 ± 46.38)		12.66 (2.9 ± 55.86)	
Specific treatment				
Yes	1	0.09	1	0.04*
No	2.83 (0.83 ± 9.61)		3.6 (1.03 ± 12.54)	
Serum IGF1 levels				
Q1 (lowest)	6.1 (4.7 ± 8.42)	0.002*	8.43 (2.4 ± 4.9)	< 0.001*
Q2	8.9 (4.8 ± 16.6)	0.003*	3.01 (0.3 ± 1.2)	0.03*
Q3	2.3 (0.6 ± 18.08)	0.14	2.9 (0.59 ± 1.4)	0.7
Q4 (highest)	Reference		Reference	

*Significant

*MFIS* modified fatigue impact scale, *IGF-1* insulin-like growth factor 1, *RR* relapsing-remitting, *EDSS* expanded disability status scale, *Q1* ≤ 90 ng/mL, *Q2* 90–120 ng/mL, *Q3* 121–190 ng/mL, *Q4* ≥ 191 ng/mL

Among the patient group, IGF-1 serum level was significantly lower in patients with MoCA score ≤ 26, MFIS score ≥ 38, those with EDSS (6–9.5), progressive MS, and duration of illness > 5 years. However, serum IGF1 levels in MS patients without specific treatment were lower than those on specific treatment but with no statistical difference (Table 3).

**Table 3:** Relationship between serum IGF-1 levels and clinical scales and clinical parameters as well as treatment among multiple sclerosis patients

Variable		Serum IGF levels (M ± SD)	*P* value
Cognitive affection (MoCA scale score)			
> 26		189 ± 22.96	< 0.001*
≤ 26		91.96 ± 21.20	
Fatigue scale (MFIS)			
≥ 38		103.09 ± 38.59	< 0.001*
< 38		180.38 ± 35.74	
EDSS			
0–5.5		176.2 ± 37.26	0.001*
6–9.5		89.2 ± 22.05	
Disease course			
Progressive	12	81.4 ± 22.03	< 0.001*
Relapsing remittent	34	155.4 ± 49.06	
Disease duration			
≤ 5 years		153.68 ± 51.94	0.004*
> 5 years		107.4 ± 43.47	
Specific treatment			
Yes		159.89 ± 49.12	1.0
No		124.54 ± 52.63	

*Significant

*EDSS* expanded disability status scale, *IGF-1* insulin-like growth factor 1, *MFIS* modified fatigue impact scale, *MoCA* Montreal cognitive scale

**Fig. 1:**
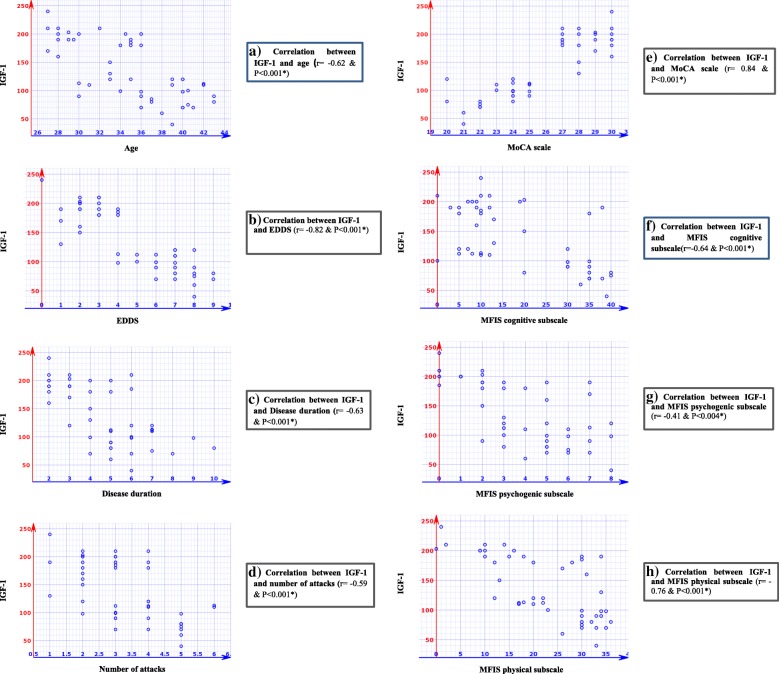
Correlation coefficient of variables with serum levels of IGF-1 in MS patients

A significant negative correlation was detected between serum IGF-1 levels and age, EDSS, disease duration, relapse rate, and MFIS score (*P* < 0.001). But a highly significant positive correlation was detected between serum IGF-1 levels and MoCA score (*P* < 0.001) (Fig. 1).

## Discussion

Insulin-like growth factor 1 (IGF-1) is a potent neuroprotective factor for neurons involved in oligodendrocyte precursor cell proliferation and differentiation as well as in myelin synthesis (Ghassan et al. 2017). As both of cognitive impairment and fatigue can affect up to 70% of MS patients, we conducted this study searching for a possible association between serum IGF-1 levels on the one hand and cognition impairment as well as fatigue on the other hand.

The present study showed that MS patients have a statistically significant higher MFIS score (physical, cognitive, and psychosocial) and a statistically significant low MoCA score than the controlled group. This finding met with those of Pellicano and colleagues (2010) and Freitas and colleagues (2018) respectively.

For data regarding MS patients with cognitive impairment (MoCA score < 26), they had significantly older age, progressive disease course, higher expanded disability status scale (EDSS), longer disease duration, and higher relapse rate than MS patients with normal cognitive function (MoCA score ≥ 26). Our results are supported by that of Aksoy and colleagues (2013) who found that cognitive impairment was significantly higher in advanced age. Also, Ashrafi and colleagues (2016) found that those with cognitive impairment had significantly higher disease duration and higher EDSS.

In regard to the fatigue in MS patients, this study demonstrated that fatigue was statistically significant with higher age, progressive disease course, higher EDSS, longer disease duration, and higher relapse rate when compared with those MS patients without fatigue. In line with the present study, Pellicano and colleagues (2010) found that in MS patients, there was a significant relationship between EDSS and fatigue severity.

According to our results, serum IGF-1 levels in MS patients have no significant difference than those in control group (*P* = 0.19). Our results corroborate the findings of Gironi et al. (2013) and Lanzillo et al. (2011); they showed no significant difference between MS patients and the control group. Also, Wilczak et al. (1998) found no difference in serum or CSF of IGF-1 between MS patients and controls. However, Ghassan and colleagues (2017) reported that there was a significant increase in serum IGF-1 levels in MS patients when compared to controls but this difference in results could attribute to the change in mean of age and disease duration. The increase in specific components of the peripheral IGF system may be primarily associated with the early stages of MS (Hosback et al. 2007).

In our patient group, serum IGF-1 levels were significantly low in patients with primary progressive MS and those with high EDSS ≥ 6 and with disease duration > 5 years also; IGF-1 showed a significant negative correlation with EDSS, disease duration, and number of attacks suggesting the presence of correlation of serum IGF-1 levels and accumulation of disability in MS. This result is in agreement with a previous study by Akcali et al. (2017).

IGF-1 is involved in the regulation of the immune system, oligodenerocyte proliferation, and survival (El-Tamawy et al. 2016). Fedorishin et al. (2017) evaluated the effect of IGF-1 on demyelination and reported that IGF-1 treatment reduce the clinical deficits, size, and number of lesion.

In this study, serum IGF-1 has no significant difference in patients who received specific treatment for MS and those without specific treatment. This is in parallel to Lanzillo et al. (2017) who reported that serum IGF-1 levels were not significantly modified after 1 and 2 years of treatment by interferon β (INF-β). On the other side, Hosback et al. (2007) observed an increase of serum IGF-1 levels in MS patients on IFN-β therapy compared to untreated MS patients, but high percentages of our patients were not receiving any specific immune modulation treatment which should be considered.

According to this study, IGF-1 has a negative correlation with the age of patients (*p* < 0.001) similar to those of Akcali et al. (2017) and Lanzillo et al. (2017).

Regarding cognitive function, we found that IGF-1 was significantly lower in MS patients with cognitive impairment than MS patients with normal cognitive function. Also, IGF-1 has a significant positive correlation with MoCA score performance and low level of IGF-1 also was found as a risk factor for cognitive impairment in MS patients. These results are parallel to those of Angelini and colleagues (2009) who found that serum IGF-1 level decline is parallel to age-dependent cognitive impairment and dementia. They also reported that serum IGF-1 level lower than 79.4 μg/L seems to be a biomarker for cognitive function.

Serum IGF-I showed significant positive correlation with cognitive deterioration in Alzheimer’s disease (AD) (Kimoto et al. 2016; Doi et al. 2015). Moreover, increasing serum IGF-1 levels were reported to improve cognition in those at high risk for AD (Baker et al. 2012). In addition, associations between serum IGF-1 levels and cognitive function in patients with growth hormone deficiency (Webb et al. 2012; Licht et al. 2014), infantile spasms (Riikonen et al. 2010), Parkinson’s disease (Pellecchia et al. 2013), and delirium (Adamis and Meagher 2011) were reported.

In a longitudinal study on 1318 elderly people, Dick et al. (2003) have founded that IGF-1 value was directly related to the speed of information processing, memory, and mini-mental state examination (MMSE) score. Also IGF-1 has a significant positive correlation with executive function (Al-Delaimy et al. 2009) and global cognitive function (Erickson and Barnes 2003).

The brain areas pivotal for cognition are the temporal lobe (hippocampus and para hippocampus areas) and prefrontal cortex which are normally rich in IGF-1 receptors (Licht et al. 2014). With aging, there is a significant reduction in both serum level of IGF-1 and its receptors in these areas with possible secondary decline in cognitive function (Lai et al. 2000). Calabrese et al. (2010) demonstrated more thinning in the fronto-temporal prefrontal cortices in mild and severe cognitive impairment MS patients.

According to fatigue, the present study showed a significant negative correlation between serum IGF-1 levels and MFIS results; also, low serum IGF-1 levels were found to be a risk factor for fatigue. In the same line with our results, Allain et al. (1997) reported lower serum levels of IGF-1 in chronic fatigue syndrome compared to those in healthy controls. Also, Bjersing et al. (2013) found evidence of a positive role for IGF-1 on fatigue in patients with fibromyalgia. A higher level of IGF-1 has associated with better physical fitness among both young (Nindl et al. 2011) and older individuals (Taekema et al. 2011).

IGF-1 is a hormone stimulated by the growth hormone, particularly during deeper “slow-wave” sleep (Ursavas et al. 2007). Total sleep deprivation (25 h of continuous wakefulness) in healthy young men is transiently associated with a significant decrease in circulating free IGF-I concentrations that are restored after one night of recovery (Chennaoui et al. 2014). Sleep disorders and obstructive sleep apnea (OSA) may affect the growth hormone/insulin-like growth factor 1 (GH/IGF-1) axis with an inverse relationship between IGF-1 plasma concentration and the severity of OSA (Ursavas et al. 2007).

Fatigued MS patients are more likely to have disrupted sleep. Several sleep disorders are reported to be common in the MS population such as sleep-related breathing disorder, insomnia, rapid eye movement sleep behavior disorder, narcolepsy, restless leg syndrome, and circadian sleep rhythm disorders. Moreover, sufficient treatment of an underlying sleep disorder may improve fatigue (Braley and Chervin 2010).

## Conclusions

A lower level of serum IGF-1 levels in MS patients presented with fatigue and cognitive impairment was reported. We suggest that serum IGF-I levels should not be considered as biomarkers of disease, but rather prognostic biomarkers. These findings should encourage larger studies to dissect further the involvement of IGF-I in mechanisms of fatigue and cognitive impairment MS patients.
